# Unusual coexistence of low-grade mucinous cystic neoplasm and idiopathic granulomatous hepatitis

**DOI:** 10.1016/j.ijscr.2024.110367

**Published:** 2024-09-30

**Authors:** Faten Limaiem, Aziz Atallah, Sadok Megdiche, Oumayma Cherif, Zied Hadrich Sahir Omrani

**Affiliations:** a University of Tunis El Manar, Faculty of Medicine of Tunis, Tunisia; b Pathology Department, Mongi Slim Hospital La Marsa, Tunisia; c Department of Surgery, Mongi Slim Hospital La Marsa, Tunisia

**Keywords:** Mucinous cystic neoplasm, Liver, Idiopathic granulomatous hepatitis, Pathology, Surgery

## Abstract

**Introduction and importance:**

Mucinous cystic neoplasms (MCNs) of the liver are rare precancerous lesions, accounting for less than 5 % of all hepatic cysts. The coexistence of MCNs with idiopathic granulomatous hepatitis is exceedingly uncommon and lacks documentation in the existing literature.

**Case presentation:**

A 43-year-old Tunisian woman with no significant medical history presented with persistent right hypochondrium pain. Clinical examination revealed a palpable mass in the right hypochondrium. Laboratory tests indicated elevated liver enzymes and cholestasis. Imaging studies identified an 18 × 11 cm cystic formation in the right liver lobe, initially suspected to be a type I hydatid cyst. The patient underwent a right hepatectomy, and histological examination confirmed the presence of a low-grade MCN of the liver coexisting with idiopathic granulomatous hepatitis. The patient experienced an uncomplicated postoperative recovery.

**Clinical discussion:**

In our case, the concomitant presence of MCN and idiopathic granulomatous hepatitis was incidental, without any causal link. The definitive diagnosis of these two conditions relies on histopathological examination. It is essential to surgically remove the MCN and identify the cause of granulomatous hepatitis to effectively manage the patient.

**Conclusion:**

This case underscores the uncommon occurrence of both MCN and idiopathic granulomatous hepatitis in the liver, highlighting the diagnostic challenges associated with the latter. Accurate diagnosis through thorough evaluation is essential for effective management.

## Highlights

• The co-occurrence of hepatic mucinous cystic neoplasm and idiopathic granulomatous hepatitis in the liver is exceptionally rare with limited medical documentation.
• Recognizing rare liver conditions emphasizes the necessity of a multidisciplinary approach for accurate diagnosis and effective treatment.
• Healthcare providers should watch for unusual liver lesion presentations, consider coexisting conditions, and prioritize precise diagnosis and tailored treatment for optimal care.
• In our case, the coexistence of mucinous cystic neoplasm and idiopathic granulomatous hepatitis was incidental, with histopathological examination being crucial for diagnosis.

## 1 Introduction

Hepatic cystic lesions encompass a diverse range of diseases with distinct etiologies, clinical presentations, and treatment approaches [1]. Among them, intrahepatic biliary mucinous cystic neoplasms (MCNs) are relatively uncommon, comprising approximately 5–10 % of biliary-origin intrahepatic cystic lesions [[1], [2], [3], [4]] and around 1 % of all hepatic cystic lesions [5,6]. Precise preoperative diagnosis of MCNs can be difficult due to their resemblance to other liver lesions, posing a diagnostic challenge. Granulomatous hepatitis can arise from infectious, non-infectious, autoimmune, or drug-induced causes, and in certain cases, it may be idiopathic with no identifiable underlying etiology [7]. However, the coexistence of MCNs and granulomatous hepatitis has not been previously reported and requires histological examination of the surgical specimen for confirmation. This paper presents an intriguing case of a female patient with no significant medical history, who presented with both intrahepatic MCN and idiopathic granulomatous hepatitis. This unusual occurrence underscores the rarity of encountering both conditions simultaneously.

This case report has been reported in line with the SCARE Criteria [8].

## 2 Case description

**Fig. 1:**
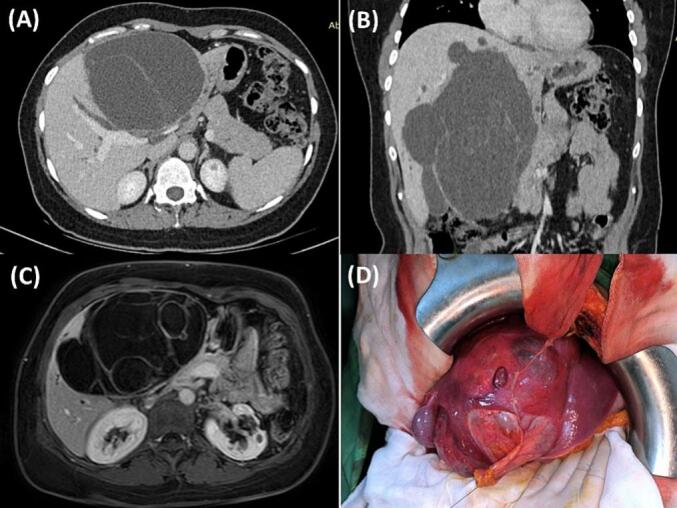
A and B: Abdominal Computed Tomography Scan illustrating a 17 cm multiloculated cystic mass with exophytic growth, compressing the biliary ducts, portal bifurcation, and inferior vena cava. C: Magnetic Resonance Imaging showing a multiloculated cystic mass, on high T2 signal, low T1 signal, and enhanced septa post-Gadolinium. D: Intraoperative view revealing a 17 cm multilobulated cystic mass straddling segments IV and V.

**Fig. 2:**
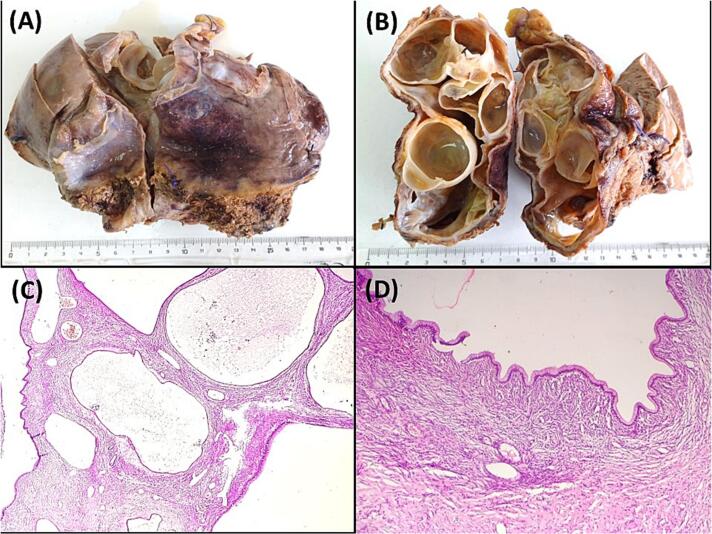
A and B: Macroscopic view of low-grade mucinous cystic neoplasm, highlighting its distinct multilocular configuration. Cut section of the cyst, revealing the well-defined multilocular structure of the hepatic mucinous cystic neoplasm. C: Multiloculated hepatic cyst with columnar epithelial cells and ovarian-type stroma, **(**Hematoxylin and eosin, magnification × 100). D: Histological analysis of low-grade mucinous cystic neoplasm. Columnar biliary-type epithelium overlying dense spindled ovarian-type stroma, **(**Hematoxylin and eosin, magnification × 100).

**Fig. 3:**
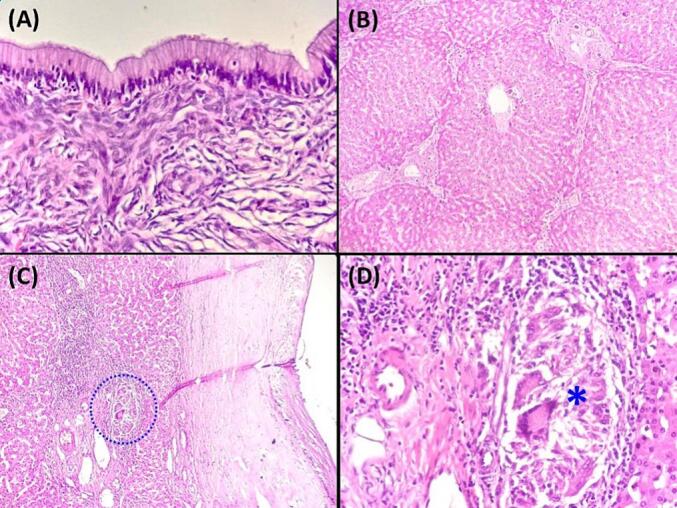
A: The mucinous cystic neoplasm exhibits tall columnar mucinous epithelium lining the cyst, displaying small basally located nuclei. The epithelium overlies a distinctive ovarian-type stroma composed of densely packed spindle cells with round to oval nuclei and scant cytoplasm. **(**Hematoxylin and eosin, magnification × 400). B: Nodular Regenerative Hyperplasia **(**Hematoxylin and eosin, magnification × 100). C: The figure highlights the presence of a clustered granuloma within a portal tract, indicated by a blue discontinuous line encircling the area **(**Hematoxylin and eosin, magnification × 100). (For interpretation of the references to colour in this figure legend, the reader is referred to the web version of this article.) D: The granuloma is composed of multinucleated giant cells and epithelioid cells. (Blue asterisk) **(**Hematoxylin and eosin, magnification × 400). (For interpretation of the references to colour in this figure legend, the reader is referred to the web version of this article.)

A 43-year-old Tunisian woman with an unremarkable medical history presented with persistent right hypochondrium pain over a four-month period. The patient denied experiencing anorexia or weight loss. Upon clinical examination, she displayed a good general condition, with a performance status (PS) of 0 indicating excellent overall functional health. The patient had a BMI of 29 kg/m^2^ and a non-tender 10 cm mass in the right hypochondrium. Laboratory tests showed cytolysis three times the normal and cholestasis four times the normal. Hydatic serology was negative, and tests for carcino-embryonic antigen (CEA), carbohydrate antigen 19–9 (CA 19–9), and α-fetoprotein were normal. Abdominal ultrasound identified an 18 × 11 cm cystic formation in the right liver lobe, suggestive of a type I hydatid cyst. CT imaging detailed a 17 cm multiloculated cystic mass with exophytic development, compressing the biliary ducts, portal bifurcation, and inferior vena cava, with no other lesions detected (Fig. 1A and B). Ascites was not observed. MRI findings supported a diagnosis of multiloculated cystadenoma or cystadenocarcinoma, based on high T2 signal, low T1 signal, and enhanced septa post-Gadolinium (Fig. 1C). A percutaneous liver biopsy was not carried out. Surgery was performed via a Makuchii incision, revealing a 17 cm multilobulated cystic mass straddling segments IV and V, resembling a hepatic cystadenoma (Fig. 1D). A right hepatectomy was performed. Gross examination of the hepatic mass revealed a cystic, multiloculated structure with a smooth, glistening white-tan lining, filled with clear fluid (Fig. 2A, B). Histological examination showed columnar biliary-type epithelium overlying a dense spindled stroma resembling ovarian tissue. No nuclear pseudostratification or mitotic figures were observed (Figs. 2C, D, 3A). Notable sheets of foamy histiocytes and foreign body granulomas were present within the cyst wall. The surrounding liver parenchyma displayed nodular regenerative hyperplasia, which manifested as the development of liver nodules interspersed with areas of atrophy, without the presence of fibrous septa. (Fig. 3B), and the portal and periportal areas showed numerous well-formed collections of epithelioid histiocytes known as epithelioid granulomas, accompanied by multinucleated giant cells and lymphocytes (Fig. 3C, D). Patchy inflammation was observed within the hepatic lobules, while fibrinoid necrosis or caseous necrosis were absent. Sarcoidosis and tuberculosis were ruled out based on laboratory tests, imaging, and clinical examination. In this case, laboratory investigations yielded normal results for specific sarcoidosis tests, indicating the absence of typical disease markers. Imaging studies, encompassing chest X-rays and CT scans, did not reveal anticipated tuberculosis features like cavitations or nodules, nor did they show sarcoidosis indicators such as bilateral hilar lymphadenopathy. Clinical examinations were meticulously performed to pinpoint key symptoms distinctive to these conditions, all of which were notably absent in the patient. The collective results, bolstered by negative biopsies of the accessory salivary glands devoid of granulomas or acid-fast bacilli, along with negative microbiological cultures, presented compelling evidence for confidently excluding tuberculosis and sarcoidosis in this clinical scenario. Therefore, the final diagnosis was a low-grade MCN of the liver associated with idiopathic granulomatous hepatitis. The postoperative recovery was smooth, with the resolution of abdominal pain, leading to the patient's discharge on the sixth day after surgery. During the two-month follow-up, the patient was well and free of symptoms.

## 3 Discussion

MCN, a cystic epithelial neoplasm, typically arises within the liver parenchyma, with the right lobe being more commonly affected (55 %) [4]. While accounting for 80–90 % of cases, it can also manifest in the extrahepatic biliary tree and the gallbladder [9]. The exact etiology of MCN remains unclear. This neoplasm, prevalent in women aged forty to fifty, rarely occurs in children [10]. MCN tends to grow slowly, with sizes typically varying from 1.5 cm to 35 cm in diameter [9]. The MCN in our case had a diameter of 17 cm. Due to their slow growth, a significant proportion of MCN remain asymptomatic. However, symptomatic cases often present with abdominal swelling and, in some instances, abdominal pain or discomfort resulting from pressure effects, as observed in our case. Occasionally, an abdominal mass can be identified during physical examination [6]. In our case, a non-tender mass was discovered in the right hypochondrium. Markers with high specificity for hepatic MCN have not been definitively established. Although CA19–9 is occasionally used as a biomarker, its levels may not consistently increase in affected patients and can be normal in some cases of hepatic MCN [4,6]. In our case, the results of CEA, CA 19–9, and α-fetoprotein tests were within normal limits. Ultrasound and CT scans are frequently employed as the primary diagnostic methods for evaluating cystic liver lesions. Ultrasound imaging can often show echo-lucent cystic lesions with clear boundaries and intricate internal structures. On the other hand, CT scans might detect a liver mass characterized by low density, internal partitions, and nodules. These lesions may display enhancement along their walls and internal septa [11,12]. MRI typically displays multilocular hyperintensity on T2-weighted images, characterized by a well-defined internal septum and variable signal intensity on T1-weighted images. Malignancy indicators include increased thickening and enhancement of the septa, nodules, and capsule, along with the presence of coarse calcifications. The management approach for non-invasive MCN differs significantly from that of other non-neoplastic cysts. Complete surgical resection is the preferred treatment option whenever feasible, considering the potential for malignant transformation in up to 15 % of patients [4,6,11,12]. The surgical technique should be tailored to the tumor's location and the patient's characteristics. Histologic examination is essential for the precise diagnosis of MCN in the liver. MCNs typically exhibit cysts lined with epithelial cells, which can be columnar, cuboidal, or flattened, arranged in a single row. Polypoid or papillary projections may occasionally be present within these cysts. Immunohistochemistry analysis demonstrates positive staining for CK7, CK19, CK8, CK18 (in biliary areas), or mucicarmine (in mucinous areas) in the epithelium of MCNs. [9,13]. According to the 2019 WHO classification, MCNs are classified using a two-tiered system based on the evaluation of cytoarchitectural abnormalities [9]. This system distinguishes between low-grade MCN and high-grade MCN, with the highest degree of cytoarchitectural abnormalities being the determining factor [9]. Thorough sampling is crucial in cases of MCNs due to extensive denudation and the potential coexistence of varying degrees of dysplasia. Liver granulomas can vary in prevalence, ranging from 1 % to 15 % [14]. They can be detected incidentally in asymptomatic individuals or indicate granulomatous hepatitis, which can lead to liver failure or cirrhosis in chronic cases. The causes of liver granulomas vary globally, with mycobacterial infections prevalent in developing countries and autoimmune diseases like primary biliary cirrhosis and sarcoidosis common in Western populations [7,14]. Other causes include schistosomiasis, drug-related granulomas, and idiopathic cases. Although available techniques exist, initial diagnosis remains challenging, with approximately 36 % of patients remaining undiagnosed [7,14]. A comprehensive evaluation involving medical history, physical examination, and liver histology is vital for accurately determining the underlying cause [7,14]. Factors such as travel history, endemic regions for infections, autoimmune disease or immunodeficiency history, and medication use can provide valuable clues. In the specific case discussed, histological examination of the liver revealed multiple portal and periportal granulomas without evidence of foreign bodies, microorganisms, or parasitic infections. No history of medication use, personal or family history of autoimmune disease or immunodeficiency was noted. Laboratory tests and imaging techniques effectively ruled out tuberculosis and sarcoidosis. Consequently, the granulomatous liver disease in this case was classified as idiopathic.

In conclusion, this case report brings to light the rare coexistence of low-grade MCN and idiopathic granulomatous hepatitis in the liver. The simultaneous occurrence of MCN and idiopathic granulomatous hepatitis in our case was incidental, lacking any causal connection. Accurate diagnosis of these conditions relies on histological examination, with surgical intervention recommended for MCNs to reduce recurrence and malignant transformation risks. Post-resection monitoring is crucial for detecting potential issues. The strengths of this case lie in illuminating this clinical rarity and emphasizing the importance of thorough evaluation and histological examination. However, limitations include sparse data, hindering in-depth analysis, and challenges in generalizing findings to broader contexts. Incomplete follow-up data and varying treatment responses among similar cases also impact the identification of optimal management strategies.

## Consent statement

Written informed consent was obtained from the patient for publication of this case report and accompanying images. A copy of the written consent is available for review by the Editor-in-Chief of this journal on request.

## Provenance and peer review

Not commissioned, externally peer-reviewed.

## Ethical approval

Ethical approval for this study was provided by the Ethical Committee of Mongi Slim University Hospital, Marsa, Tunisia.

## Funding

This research did not receive any specific grant from funding agencies in the public, commercial, or not-for-profit sectors.

## Author contribution

**Dr. Faten LIMAIEM**: Prepared, organized, wrote, and edited all aspects of the manuscript.

Performed the gross and microscopic pathologic evaluation of the pathology specimen.

**Dr. Aziz ATALLAH**, **Sadok Megdiche, Oumayma Cherif, Zied Hadrich, Sahir Omrani**: Read, edited, and approved the final version of the manuscript. Contributed to data acquisition, analysis, and interpretation. Provided final approval of the manuscript before its submission.

## Guarantor

Dr. Faten LIMAIEM.

## Research registration number

N/A.

## Conflict of interest statement

None declared.

## References

[1] Klompenhouwer A.J., Ten Cate D.W.G., Willemssen F.E.J.A., Bramer W.M., Doukas M., de Man R.A. (Oct 2019). The impact of imaging on the surgical management of biliary cystadenomas and cystadenocarcinomas; a systematic review. HPB (Oxford).

[2] Jwa E.K., Hwang S. (2017). Clinicopathological features and postresection outcomes of biliary cystadenoma and cystadenocarcinoma of the liver. Ann. Hepatobiliary Pancreat. Surg..

[3] Yoshida N., Mitsufuji S., Okuda T., Yasukawa S., Sakagami J., Wakabayashi N. (2004). Biliary cystadenocarcinoma from biliary cystadenoma. Nihon Shokakibyo Gakkai Zasshi.

[4] Hutchens J.A., Lopez K.J., Ceppa E.P. (2023). Mucinous cystic neoplasms of the liver: epidemiology, diagnosis, and management. Hepat. Med..

[5] Sang X., Sun Y., Mao Y., Yang Z., Lu X., Yang H. (2011). Hepatobiliary cystadenomas and cystadenocarcinomas: a report of 33 cases. Liver Int..

[6] El-Magd El-Sayed Abou, El-Shobari Mohamed, Abdelsalam Ramy A., Abbas Amr, Elmahdy Youssif, Hamed Hosam (2023). Clinicopathological features and management of biliary cystic tumors of the liver: a single-center experience. Langenbecks Arch. Surg..

[7] Gaspar R., Andrade P., Silva M., Peixoto A., Lopes J., Carneiro F. (2018). Hepatic granulomas: a 17-year single tertiary Centre experience. Histopathology.

[8] Sohrabi C., Mathew G., Maria N., Kerwan A., Franchi T., Agha R.A. (2023). The SCARE 2023 guideline: updating consensus surgical CAse REport (SCARE) guidelines. Int. J. Surg..

[9] Basturk O.N.Y., Aishima S., Esposito I., Klimstra D.S., Komuta M., Zen Y. (2019).

[10] Bezabih Y.S., Tessema W.A., Getu M.E. (2021). Giant biliary mucinous cystadenoma mimicking mesenchymal hamartoma of the liver in a child: a case report. Int. J. Surg. Case Rep..

[11] Alzoubi M.N., Alhendi R.B., Eyalawwad A.A. (2022). Liver mucinous cystic neoplasm with obstructive jaundice. Cureus.

[12] Soochan D., Keough V., Wanless I., Molinari M. (2012). Intra and extrahepatic cystadenoma of the biliary duct. Review of literature and radiological and pathological characteristics of a very rare case. BMJ Case Rep..

[13] Devaney K., Goodman Z.D., Ishak K.G. (1994). Hepatobiliary cystadenoma and cystadenocarcinoma. A light microscopic and immunohistochemical study of 70 patients. Am. J. Surg. Pathol..

[14] Mironova M., Gopalakrishna H., Rodriguez Franco G., Holland S.M., Koh C., Kleiner D.E., Heller T. (Mar 18, 2024). Granulomatous liver diseases. Hepatol. Commun..

